# Relationship between right atrium area and right ventricular ejection fraction on magnetic resonance imaging: comparison with other prognostic markers in patients with pulmonary arterial hypertension

**DOI:** 10.1590/0100-3984.2018.0128

**Published:** 2019

**Authors:** Marcelo M. Mello, Guilherme Watte, Stephan Altmayer, Yana L. R. Pallaoro, Fernanda B. Spilimbergo, Daniela C. Blanco, Gisela M. B. Meyer, Edson Marchiori, Bruno Hochhegger

**Affiliations:** 1 Pulmonary Hypertension Group, Irmandade Santa Casa de Misericórdia de Porto Alegre, Porto Alegre, RS, Brazil.; 2 Medical Imaging Research Laboratory, Federal University of Health Sciences of Porto Alegre (UFCSPA), Porto Alegre, RS, Brazil.; 3 School of Medicine, Graduate Program in Medicine and Health Sciences, Pontifical Catholic University of Rio Grande do Sul (PUCRS), Porto Alegre, RS, Brazil.; 4 Federal University of Rio de Janeiro (UFRJ), Rio de Janeiro, RJ, Brazil.

**Keywords:** Hypertension, pulmonary, Magnetic resonance imaging, Cardiac catheterization, Echocardiography

## Abstract

**Objective:**

To compare the right atrium (RA) area and right ventricular ejection fraction (RVEF) with other known prognostic markers in patients with pulmonary arterial hypertension (PAH).

**Materials and Methods:**

This was a retrospective study of 74 patients diagnosed with PAH by right heart catheterization at a referral center between January 2018 and May 2018. All of the patients underwent cardiac magnetic resonance imaging (MRI) within 3 months of the right heart catheterization (RHC), as well as undergoing echocardiography, a 6-minute walk test, and determination of the level of N-terminal pro-brain natriuretic peptide (NT-proBNP) within a month of the RHC. We attempted to determine whether the cardiac MRI-derived RA area correlated with ions between RVEF and RA area measured by that determined by echocardiography, as well as whether the cardiac MRI-derived RA area and RVEF correlated with the 6-minute walk distance and NT-proBNP level.

**Results:**

The MRI-derived RA area demonstrated a weak correlation with the pulmonary vascular resistance measured by RHC (*r* = 0.268; *p* = 0.055) and a moderate correlation with the NT-proBNP (*r* = 0.429; *p* = 0.003). All correlations between clinical characteristics and the RVEF were statistically significant. In the univariate linear analysis, the RVEF showed stronger correlations with the clinical characteristics than did the RA area.

**Conclusion:**

In patients with PAH, cardiac MRI-derived RVEF appears to correlate more strongly with other prognostic factors than does RA area.

## INTRODUCTION

Pulmonary arterial hypertension (PAH) is a progressive disease of the pulmonary vasculature that can involve multiple clinical conditions^(1)^. It is defined as a mean pulmonary arterial pressure ≥ 25 mmHg at rest and a pulmonary artery wedge pressure ≤ 15 mmHg, measured by right heart catheterization (RHC), as previously described^(2)^. The clinical progression of the disease is characterized by impaired exercise tolerance, right ventricular (RV) hypertrophy, right-sided heart dilatation, and cardiac failure^(3)^.

Increasingly, researchers are looking for prognostic markers to assess risk in PAH^(4)^. Recent studies have shown that the symptoms and outcomes of PAH are largely related to RV function^(5)^. Echocardiography is currently the image modality most widely used in evaluating the structure and function of the RV^(6)^. However, echocardiography is operator dependent and the image quality is often inadequate, especially for the RV^(7)^. Therefore, evaluation of the RV ejection fraction (RVEF) through the use of cardiac magnetic resonance imaging (MRI) has emerged as the gold standard for the assessment of RV function^(8)^.

In many chronic diseases, such as PAH, risk assessment is essential to improve the management of cases^(1,9)^. According to the European Society of Cardiology/European Respiratory Society (ESC/ERS) pulmonary hypertension guidelines, risk should be assessed every 3-6 months and should involve multiple parameters, in order to evaluate disease progression and the response to treatment^(1,10)^. As per the ESC/ERS guidelines, the only imaging parameter recommended routinely is the area of the right atrium (RA). However, assessment of the RVEF by cardiac MRI is known to have better prognostic value in PAH^(11,12)^. Therefore, this study aims to evaluate the RA area and RVEF in comparison with other prognostic parameters in patients with PAH.

## MATERIALS AND METHODS

### Patients

This was a retrospective study in which we identified 74 patients diagnosed with PAH by RHC at a referral center between January 2018 and May 2018. All of the patients underwent cardiac MRI within 3 months of the RHC, as well as undergoing echocardiography, a 6-minute walk test, and determination of the level of N-terminal pro-brain natriuretic peptide (NT-proBNP) within a month of the RHC.

### Cardiac MRI protocol

Cardiac MRI was performed in a 1.5 T scanner (Magnetom Aera; Siemens, Erlangen, Germany). A half-Fourier acquisition single-shot turbo spin-echo sequence was used, and the field-of-view (FOV) was patient adapted. The following sequence parameters were used: repetition time/echo time (TR/TE), infinite/92 ms; flip angle, 150º; parallel acquisition factor, 2; slice thickness, 4 mm; distance factor, 20%; matrix, 380 × 256 (transverse plane) or 400 × 320 (coronal plane); and mean acquisition time, 90 s. The following measures were used for four-chamber and short-axis cine images: electrocardiogram-gated steady-state free-precession sequence; rate, 20 frames/cardiac cycle; slice thickness, 5 mm; FOV, 280-320 × 280-400 mm; matrix 256 × 256; bandwidth, 125 kHz/pixel; and TR/TE, 3.7/1.6 ms. The phase-contrast sequence was acquired orthogonal to the pulmonary artery trunk with a TR/TE of 5.6/2.7 ms, a slice thickness of 10 mm, an FOV of 280-320 × 280-400 mm, and a matrix of 256 × 128.

### Imaging analysis

**Figure 1:**
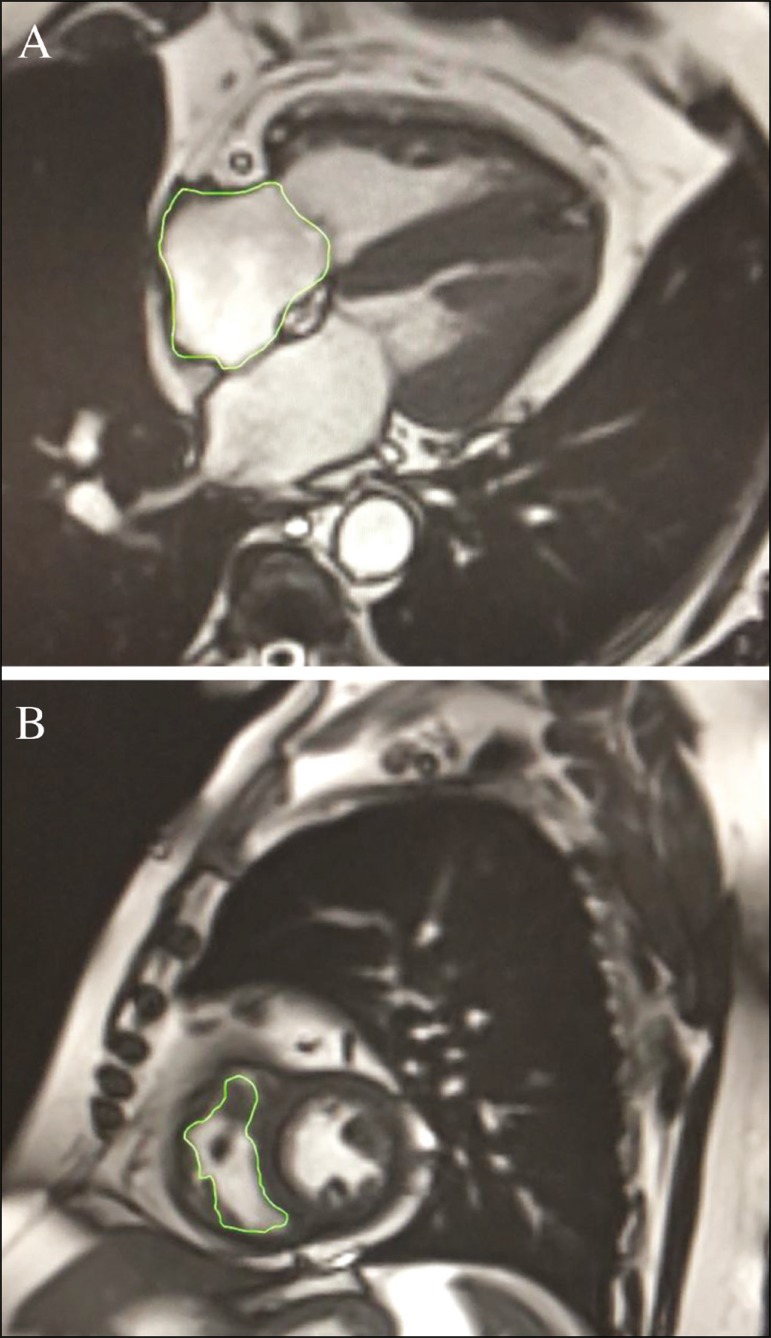
Example of segmentation of the RV area at its maximum diameter in the four-chamber view (**A**) and in the short-axis view (**B**).

All cardiac MRI acquisitions were reviewed by two chest radiologists (with 8 and 10 years of experience in cardiac MRI, respectively) who were blinded to the RHC hemodynamic data. Disagreements were resolved by consensus; if a consensus could not be reached, a third radiologist (with 15 years of experience in cardiac MRI) made the final decision. The chest radiologist with 8 years of experience performed the right and left ventricular segmentation using specialized software (CardiacVX; GE Healthcare, Waukesha, WI, USA), in accordance with the latest recommendations^(13,14)^. The dimensions of the RA were measured at its maximum diameter in the four-chamber view (Figure 1A), whereas RV segmentation was performed in the short-axis view (Figure 1B).

### Statistical analysis

Data are presented as means ± standard deviations or as absolute and relative frequencies. We used chi-square tests to test associations between variables. For comparisons between continuous variables, Student’s *t*-tests or unequal variance t-tests were used. Pearson’s correlation coefficient or Spearman’s rank correlation coefficient was used in order to assess linear associations between continuous variables. Coefficients were ranked as follows: 0.00-0.19, very weak; 0.20-0.39, weak; 0.40-0.69, moderate; 0.70-0.89, strong; and ≥ 0.90, very strong^(15)^. Linear regression analysis was used in order to determine whether the demographic characteristics and interventional procedures affecting cardiac MRI were associated with the RA area and the RVEF. In all cases, values of *p* < 0.05 were considered statistically significant. All statistical analyses were performed using the Predictive Analytics Software package, version 18.0 (SPSS Inc., Chicago, IL, USA).

## RESULTS

**Figure 2:**
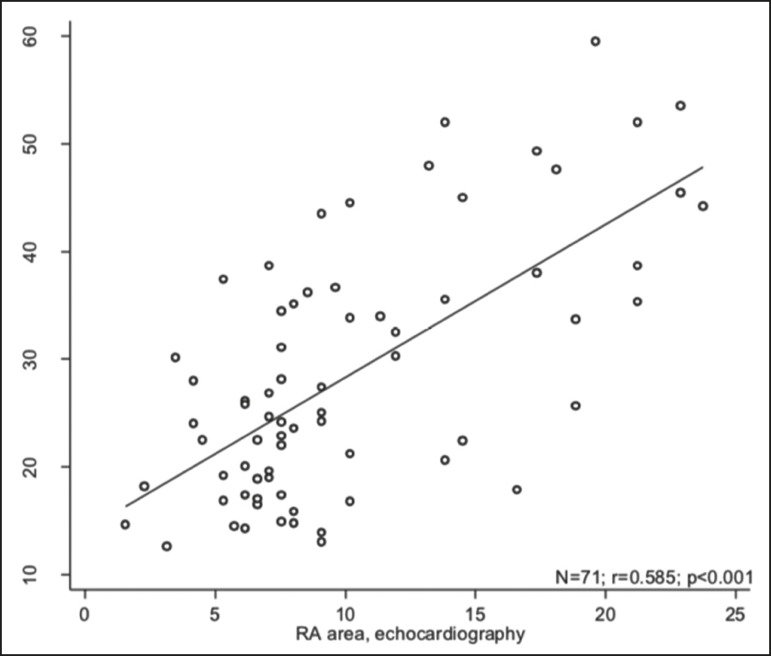
Correlation between the RA area quantified by MRI and that quantified by echocardiography (*r* = 0.585; *p* < 0.001).

The characteristics of the study subjects are described in Table 1. Correlations between clinical characteristics and cardiac MRI findings are presented in Table 2. The area of the RA demonstrated a weak correlation with pulmonary vascular resistance (PVR) measured by RHC (*r* = 0.268; *p* = 0.055) and a moderate correlation with the NT-proBNP level (*r* = 0.429; *p* = 0.003). The RVEF showed statistically significant correlations with all of the clinical characteristics evaluated (Table 2). The RA area determined by MRI demonstrated a moderate correlation with that determined by echocardiography (*r* = 0.585; *p* < 0.001), as shown in Figure 2.

**Table 1:** Demographic and clinical characteristics of the patients.

Characteristic	(N = 74)
Female, n (%)	61 (82.4)
Age (years), mean ± SD	42 ± 15
Body mass index (kg/m^2^), mean ± SD	24.2 ± 5.4
Body surface area (m^2^), mean ± SD	1.63 ± 0.2
WHO/NYHA functional class, n (%)	
I/II	47 (63.5)
III/IV	27 (36.5)
PAH etiology, n (%)	
Idiopathic	32 (43.2)
Congenital heart disease (corrected)	24 (32.4)
Connective tissue disease	14 (18.9)
HIV	3 (4.1)
Portopulmonary hypertension	1 (1.4)
Pharmacotherapy, n (%)	
None	8 (10.8)
PDE5 inhibitor	28 (37.8)
ERA	6 (8.1)
ERA + PDE-5 inhibitor	25 (33.8)
Other*	7 (9.5)
NT-proBNP (pg/mL), mean [IQR ]	238 [70-957]
RHC finding, mean ± SD	
Mean pulmonary arterial pressure (mmHg)	57.4 ± 16.6
Cardiac output (L/min)	5.1 ± 1.5
PVR (Wood units)	10.0 ± 4.8
Cardiac MRI, mean ± SD	
RA area	28.4 ± 11.6
RVEF	42.3 ± 15.2
TAPSE (mm), mean ± SD	1.58 ± 0.36
Six-minute walk test	
Six-minute walk distance (m), mean ± SD	462 ± 119
Final Borg scale score, mean [IQR]	4 [2-6]
Peripheral oxygen saturation (%), mean [IQR]	7 [1-12]
Hospitalization due to PAH in the last 12 months, n (%)	11 (14.9)
Death, n (%)	10 (13.5)

SD, standard deviation; WHO, World Health Organization; NYHA, New York Heart Association; ERA, endothelin receptor antagonist; PDE5, phosphodiesterase 5; IQR, interquartile range.

* ERA + inhaled prostanoid (n = 2); PDE-5 inhibitor + inhaled prostanoid (n = 2); ERA + PDE-5 inhibitor + inhaled prostanoid (n = 2); or soluble guanylate cyclase stimulator (n = 1).

**Table 2:** Pearson’s correlations of the RA area and of the RVEF with factors affecting cardiac MRI.

Parameter	RA area			RVEF	
	*r*	*p*		*i*	*p*
Age, years	0.128	0.277		0.269	0.030
WHO/NYHA functional class	0.020	0.867		-0.296	0.017
RHC finding					
Cardiac output, L/min	-0.196	0.120		0.297	0.025
PVR, Wood units	0.280	0.025		-0.425	< 0.001
TAPSE	-0.282	0.024		-0.443	0.001
Six-minute walk distance, m	-0.137	0.268		0.208	0.117
NT-proBNP, pg/mL	0.421	0.001		-0.548	< 0.001

WHO, World Health Organization; NYHA, New York Heart Association.

In the univariate linear analysis, the RA area correlated significantly with PVR, tricuspid annular plane systolic excursion (TAPSE), and the NT-proBNP level, as did the RVEF (Table 3). However, the RVEF also correlated significantly with age, functional class, and cardiac output.

**Table 3:** Results of the univariate regression analysis of the RA area and the RVEF in relation to factors affecting cardiac MRI.

Parameter	RA area			RVEF	
	CE (95% CI)	*p*		CE (95% CI)	*p*
Female	6.459 (−1.302, 14.220)	0.101		3.890 (−6.571, 14.552)	0.460
Age, years	0.098 (−0.067, 0.264)	0.242		0.277 (0.040, 0.514)	0.023
WHO/NYHA functional class III/IV	0.327 (−3.117, 3.771)	0.850		−6.312 (−11.442, −1.182)	0.017
RHC finding					
Cardiac output, L/min	−1.505 (−3.361, 0.349)	0.110		3.177 (0.920, 5.435)	0.007
PVR, Wood units	0.685 (0.193, 1.177)	0.007		−1.499 (−2.074, −0.923)	< 0.001
TAPSE	−9.025 (−17.342, −0.708)	0.034		17.866 (8.694, 27.038)	< 0.001
Six-minute walk distance, m	−0.013 (−0.041, 0.014)	0.333		0.027 (−0.004, 0.059)	0.095
NT-proBNP, pg/mL	0.006 (0.002, 0.009)	0.002		−0.011 (−0.015, −0.007)	< 0.001
Hospitalization due to PAH in the last 12 months	4.584 (−2.353, 11.521)	0.192		−9.918 (−22.050, 2.213)	0.107
Death	3.995 (−3.604, 11.595)	0.298		−2.283 (−14.262, 9.695)	0.705

CE, coefficient estimate; CI, confidence interval; WHO, World Health Organization; NYHA, New York Heart Association.

## DISCUSSION

Recent studies have highlighted the importance of imaging methods in the evaluation of cardiac diseases^(16-21)^. The present study compared the RVEF and RA area, as assessed by cardiac MRI, with other known prognostic markers in PAH. Our findings indicate that the RVEF and the RA area both have a relationship with the clinical prognosis. However, the RVEF showed more significant correlations and stronger associations with prognostic factors than did the RA area.

Many previous studies have attempted to identify biomarkers capable of predicting risk in patients with PAH. Such markers allow physicians to monitor disease progression, to determine the prognosis, and to assess responses to treatment in patients with PAH^(9)^. Regular risk assessment is essential in such patients^(1)^. According to the 2015 ESC/ERS pulmonary hypertension guidelines, the evaluation of multiple variables can be used in order to categorize patients with PAH as being at low, intermediate, or high risk^(1)^.

In the ESC/ERS guidelines, one of the observed parameters for risk assessment is the RA area^(1)^. The size of the RA has demonstrated prognostic value in PAH^(22,23)^. Querejeta Roca et al.^(24)^ found no correlation between RA function and hemodynamic parameters. Our findings corroborate the hypothesis that changes in the RA can manifest very late, after the RV has already failed.

In one study, the size of the RA, as determined by echocardiography, was shown to be an independent prognostic factor in patients with pulmonary hypertension^(25)^. In another study, RA size was found to be associated with a decrease in survival in patients with PAH^(26)^. Nevertheless, both of those studies measured the area of the RA by echocardiography, which is less accurate than is cardiac MRI. Nevertheless, our study demonstrated that the RVEF is a better prognostic factor than is the RA area in comparison with other biomarkers, such as TAPSE, the NT-proBNP level, and the total 6-minute walk distance.

In patients with PAH, RV function is an important predictor of survival^(13,14)^. Cardiac MRI is the gold-standard imaging modality for the evaluation of RV function, including the RVEF^(27)^, which is an established prognostic factor in patients with PAH^(12,28)^. In the present study, there was no significant association between the RVEF and mortality, likely due to the small number of events in the follow-up period.

Our study has some limitations. First, because of the retrospective nature of the study, all of the participants had previously been diagnosed with PAH, which constitutes a selection bias. Second, this was a preliminary study and involved a small number of participants. Further studies involving larger samples are needed in order to validate the results presented here. Therefore, prospective studies are warranted in order to elucidate the potential of cardiac MRI to diagnose patients referred for investigation of suspected pulmonary hypertension.

## CONCLUSION

Our study showed that the RVEF and the RA area were both associated with prognostic factors in patients with PAH. However, the RVEF showed more significant correlations and stronger associations with prognostic factors than did the RA area.
